# BENZODIAZEPINE/Z-DRUG DEPRESCRIBING DEFINITIONS AND FALLS OUTCOMES IN ELECTRONIC HEALTH RECORDS

**DOI:** 10.1093/geroni/igad104.2276

**Published:** 2023-12-21

**Authors:** Nicole Schindler, Lindsay Zepel, Matthew Maciejewski, Susan Hastings, Amy Clark, Juliessa Pavon

**Affiliations:** Duke University Medical Center, Durham, North Carolina, United States; Duke University, Durham, North Carolina, United States; Duke University, Durham, North Carolina, United States; Duke University, Durham VA, Durham, North Carolina, United States; Duke University, Durham, North Carolina, United States; Duke University School of Medicine, Division of Geriatrics, Durham, North Carolina, United States

## Abstract

Benzodiazepines and Z-drugs contribute to the burden of falls in older adults; however, there is a lack of data examining the association between deprescribing and falls in real-world settings. We explored this association in a retrospective cohort study of adults aged 65+ with chronic benzodiazepine/z-drug use who received care at an academic health system from January 2017-December 2020. Chronic use of benzodiazepine/z-drugs was defined as ≥ 3 medication dispensings and cumulative days’ supply ≥ 45 days within 100 days in 2018 based on electronic health records. Medication deprescribing was defined as having a dispensing gap of either ≥180 days or 90 days within one year of index. Non-deprescribers were matched 4:1 using propensity score methods (180-day gap sample=655; 90-day gap sample=1395). Primary outcome was time until first fall resulting in ED, inpatient, or outpatient visit during 2-year follow-up after meeting deprescriber/non-deprescriber status, modeled using a cox proportional hazards model adjusted for age, gender, and number of comorbidities and chronic medications. Using the 180-day gap deprescribing definition (n=131 deprescribers, and n=524 non-deprescribers), the cumulative incidence of falls-related acute events was 6.9% for deprescribers and 9.7% for non-deprescribers (HR 0.65 [95% CI 0.31, 1.31]). Using the 90-day-gap definition (n=279 deprescribers and n=1116 non-deprescribers), the cumulative incidence was 9.3% for deprescribers and 8.5% for non-deprescribers (HR 1.12 [0.70, 1.77]). In this small single-site sample, neither deprescribing gap definition was associated with falls. There is a need to standardize deprescribing definitions and validate them in larger studies in the context of relevant clinical outcomes.

